# Artificial Intelligence Applications in Chronic Obstructive Pulmonary Disease: A Global Scoping Review of Diagnostic, Symptom-Based, and Outcome Prediction Approaches

**DOI:** 10.3390/biomedicines13123053

**Published:** 2025-12-11

**Authors:** Alberto Pinheira, Manuel Casal-Guisande, Cristina Represas-Represas, María Torres-Durán, Alberto Comesaña-Campos, Alberto Fernández-Villar

**Affiliations:** 1 Department of Design in Engineering, University of Vigo, 36208 Vigo, Spain; acomesana@uvigo.es; 2 NeumoVigo I+i Research Group, Galicia Sur Health Research Institute (IIS Galicia Sur), SERGAS-UVIGO, 36312 Vigo, Spain; cristina.represas.represas@sergas.es (C.R.-R.); maria.luisa.torres.duran@sergas.es (M.T.-D.); jose.alberto.fernandez.villar@sergas.es (A.F.-V.); 3 Department of Computer Engineering, Superior Institute of Engineering of Porto, 4249-015 Porto, Portugal; 4 Centro de Investigación Biomédica en Red, CIBERES ISCIII, 28029 Madrid, Spain; 5 Pulmonary Department, Hospital Álvaro Cunqueiro, 36312 Vigo, Spain; 6 School of Industrial Engineering, University of Vigo, 36310 Vigo, Spain

**Keywords:** chronic obstructive pulmonary disease, artificial intelligence, machine learning, deep learning, scoping review, diagnosis, outcome prediction, symptom analysis

## Abstract

**Background**: Chronic Obstructive Pulmonary Disease (COPD) represents a significant global health burden, characterized by complex diagnostic and management challenges. Artificial Intelligence (AI) presents a powerful opportunity to enhance clinical decision-making and improve patient outcomes by leveraging complex health data. **Objectives**: This scoping review aims to systematically map the existing literature on AI applications in COPD. The primary objective is to identify, categorize, and summarize research into three key domains: (1) Diagnosis, (2) Clinical Symptoms, and (3) Clinical Outcomes. **Methods**: A scoping review was conducted following the Arksey and O’Malley framework. A comprehensive search of major scientific databases, including PubMed, Scopus, IEEE Xplore, and Google Scholar, was performed. The Population–Concept–Context (PCC) criteria included patients with COPD (Population), the use of AI (Concept), and applications in healthcare settings (Context). A global search strategy was employed with no geographic restrictions. Studies were included if they were original research articles published in English. The extracted data were charted and classified into the three predefined categories. **Results**: A total of 120 studies representing global distribution were included. Most datasets originated from Asia (predominantly China and India) and Europe (notably Spain and the UK), followed by North America (USA and Canada). There was a notable scarcity of data from South America and Africa. The findings indicate a strong trend towards the use of deep learning (DL), particularly Convolutional Neural Networks (CNNs) for medical imaging, and tree-based machine learning (ML) models like CatBoost for clinical data. The most common data types were electronic health records, chest CT scans, and audio recordings. While diagnostic applications are well-established and report high accuracy, research into symptom analysis and phenotype identification is an emerging area. Key gaps were identified in the lack of prospective validation and clinical implementation studies. **Conclusions**: Current evidence shows that AI offers promising applications for COPD diagnosis, outcome prediction, and symptom analysis, but most reported models remain at an early stage of maturity due to methodological limitations and limited external validation. Future research should prioritize rigorous clinical evaluation, the development of explainable and trustworthy AI systems, and the creation of standardized, multi-modal datasets to support reliable and safe translation of these technologies into routine practice.

## 1. Introduction

According to the most recent Global Burden of Disease study, Chronic Obstructive Pulmonary Disease (COPD) remained the third leading cause of death globally in 2023, following ischemic heart disease and stroke [1]. COPD is a respiratory condition which is characterized by constant symptoms such as dyspnea, sputum production and cough. In advanced stages of the disease, some patients may experience syncope, typically triggered by severe coughing episodes or associated cardiovascular comorbidities such as cor pulmonale [2]. These symptoms result from structural changes in the airways and alveoli that cause chronic airflow obstruction [3], which can lead to increased anxiety and depression, reduced physical activity and impaired sleep, which all result in a symptom burden [4].

Advancements in Artificial Intelligence (AI) provide innovative solutions to longstanding problems in respiratory medicine. By using machine learning (ML) and deep learning (DL), these intelligent systems can analyze complex multimodal data including imaging, spirometry, electronic health records (EHRs), wearable sensors, and patient-reported outcomes [5,6,7,8]. Regarding COPD, applications using AI have rapidly expanded, from automated interpretation of imaging and spirometry for early diagnosis [5,6], to digital health solutions for real-time symptom monitoring [7], and predictive models to predict mortality, hospitalizations and exacerbation prediction [8,9]. These approaches have shown the potential to improve clinical decision-making, personalize care, and reduce healthcare utilization.

Beyond COPD, the utility of AI and Clinical Decision Support Systems (CDSSs) has been substantiated across a spectrum of respiratory and systemic conditions [10], highlighting the transferability of these methodological advances. For instance, novel intelligent systems have been proposed to predict long-term sequelae such as dyspnea in post-COVID-19 patients [11], while unsupervised machine learning tools have proven effective in the clinical characterization of complex presentations like syncope of unclear cause [12] and Alpha-1 antitrypsin deficiency [13,14]. In parallel, advancements in oncology have led to the development of trustworthy, multi-agent AI systems that integrate imaging and tabular clinical data for the early detection of breast cancer [15], while in cardiology, Bayesian-optimized gradient boosting models are being successfully deployed to diagnose coronary heart disease in high-risk diabetic populations [16], and deep learning techniques are being applied to smartwatch-derived electrocardiograms for efficient arrhythmia detection [17]. In neurology and oncology, respectively, multimodal learning models are refining the diagnosis of Alzheimer’s disease by fusing MRI and PET data [18], while scoping reviews validate the potential of AI-supported screening to reduce radiologist workload in breast cancer programs [19]. Moreover, the integration of Large Language Models (LLMs) emerges as a critical tool for next-generation clinical decision support and healthcare administration [20]. In the domain of sleep medicine, AI-driven approaches are increasingly used for the early stratification and diagnosis of Obstructive Sleep Apnea (OSA) using clinical and demographic data, often preceding expensive polysomnography [21,22]. Furthermore, fundamental advancements in CDSS architecture, including the integration of fuzzy expert systems [23,24] and innovative data handling techniques that convert tabular clinical data into image formats for Convolutional Neural Network (CNN) analysis [25], demonstrate the growing sophistication of AI in handling heterogeneous medical data. These developments collectively reinforce the potential of AI to enhance diagnostic precision and prognostic modeling in chronic respiratory diseases.

While individual studies and reviews have explored AI applications in respiratory diseases, there is a lack of comprehensive synthesis focusing specifically on COPD across its diagnostic, symptom-based, and prognostic domains. Unlike systematic reviews, which typically aim to answer precise effectiveness questions through rigorous quality appraisal and meta-analysis, a scoping review is distinct in its objective to chart the volume, nature, and characteristics of research in a broad subject area. A scoping review is therefore timely and appropriate to map the breadth of existing evidence, clarify complex concepts regarding AI modalities, highlight methodological strengths and limitations, and identify research priorities for future clinical integration.

The application of AI in COPD is a field of exceptionally rapid growth, with a high volume of publications necessitating continuous review to map its progress [26]. While recent and valuable systematic reviews have provided critical, deep analyses of specific domains, such as meta-analyses on CT-based diagnosis [27] or long-term prognostic models [28], a comprehensive, high-level map of the entire landscape is crucial for identifying cross-domain trends, methodological gaps, and the overall state of the evidence. Conducting this review is therefore important to synthesize this heterogeneous body of work, ranging from sensor data to genomic analysis, into a coherent framework. This scoping review fulfills that need by providing a global, structured overview of AI applications across three distinct pillars: diagnosis, outcome prediction, and symptom/phenotype analysis. By offering a granular analysis of the specific AI models and data modalities being employed, this work provides a recent and holistic benchmark for researchers, clinicians, and policymakers.

## 2. Materials and Methods

### 2.1. Study Design

This scoping review was conducted following the methodological framework proposed by Arksey and O’Malley [29] and subsequently refined by Levac et al. [30], incorporating methodological guidance from the Joanna Briggs Institute (JBI) Manual for Evidence Synthesis [31]. Reporting adhered to the Preferred Reporting Items for Systematic Reviews and Meta-Analyses extension for Scoping Reviews (PRISMA-ScR) checklist.

These frameworks were selected for their suitability in systematically mapping heterogeneous and emerging fields of research, such as the application of AI in COPD. The Population–Concept–Context (PCC) framework [31], guided the development of inclusion criteria:

- Population: Patients diagnosed with COPD.
- Concept: Application of AI, including but not limited to ML and DL algorithms, for diagnosis, monitoring, symptoms evaluation or prediction of clinical outcomes.
- Context: Clinical or research setting across any demographic location.

Eligible studies included peer-reviewed journal articles and conference proceedings published in English between 2017 and 2025.

Exclusion criteria were:

- studies focused primarily on diseases other than COPD without providing COPD-specific results,
- studies that did not employ AI as a core methodological component.

### 2.2. Identification and Selection of Studies

A comprehensive literature search was conducted to identify studies investigating AI applications in COPD. Four electronic databases were searched: PubMed, Scopus, IEEE Xplore, and Google Scholar. The search strategy combined COPD-related keywords (“COPD,” “chronic obstructive pulmonary disease”) with AI-related terms (“artificial intelligence”, “machine learning”, “deep learning”). Boolean operators and database-specific subject headings were used where appropriate to ensure both sensitivity and precision.

All retrieved records were imported into the web-based version of Rayyan.ai [32], accessed between June and September of 2025, for deduplication and screening. To ensure a reliable and controlled selection process, three reviewers (A.P., M.C.-G. and A.C.-C), with domain expertise in AI methodologies, and another three reviewers (C.R.-R, M.T.-D, and A.F.-V.), with domain expertise in respiratory medicine, independently screened all titles and abstracts. This independent screening was guided by the predefined inclusion and exclusion criteria detailed in Section 2.1. Studies meeting these eligibility criteria were retrieved for full-text review. Any discrepancies between the reviewers regarding inclusion were resolved through discussion until consensus was reached, ensuring a consistent and rigorous application of the selection criteria.

The final set of publications included in the review consisted of studies that met all predefined inclusion criteria after full-text assessment.

## 3. Results

### 3.1. Study Selection

The initial database search identified 707 records. After removing 140 duplicates, 567 unique records were screened by title and abstract. A total of 395 were excluded as non-relevant, leaving 172 articles for full-text evaluation. Following detailed review, 53 studies were excluded, and 120 studies met all inclusion criteria and were incorporated into the final synthesis.

We excluded non-empirical works, such as literature reviews, editorials, and opinion pieces, as well as studies focusing on non-AI technologies or those outside the scope of COPD. Following screening, we excluded 35 articles that were outside the scope of COPD, 18 articles that AI was not a core method in this study.

**Figure 1:**
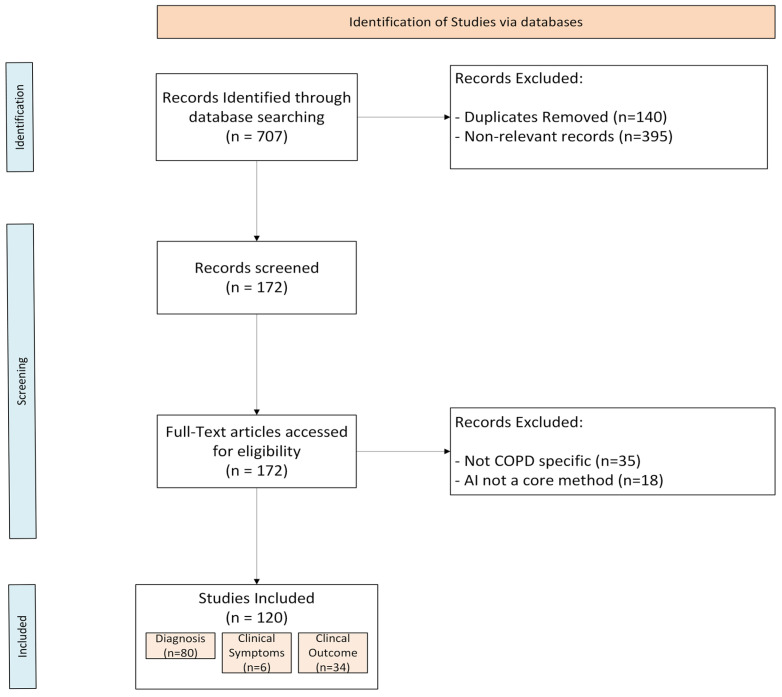
PRISMA-ScR flowchart.

A summary of the selection process is illustrated in Figure 1 (PRISMA-ScR flow diagram).

### 3.2. Diagnostic Applications of AI in COPD

A total of 80 focused on AI-based diagnostic applications of COPD, as shown in Table 1. The objectives of these studies included detecting COPD, identifying high-risk populations, staging the disease, and differentiating COPD from other respiratory conditions such as asthma, pneumonia, or adenocarcinoma [33,34,35,36].

A wide array of data modalities was utilized to train and test these models. Common data sources included chest imaging, such as CT scans and X-rays, as well as respiratory sounds, which encompassed many studies using audio data like lung sounds, cough sounds, or breath sounds. Additionally, a significant category was clinical and EHR data, which included EHRs, pulmonary function tests, and general patient records. Finally, studies also used a variety of other data types, such as genetic data, non-invasive sensor data, photoplethysmography (PPG) signals, electrocardiogram (ECG) signals, and exhaled breath samples from E-Nose sensors or other methods [36,37,38,39,40].

Geographically, the choice of data modality often reflects regional healthcare infrastructure. Studies originating from Asia contributed the highest volume, with China predominantly utilizing hospital-based EHRs and CT imaging (e.g., Lin et al. [41], Guan et al. [42], Fang et al. [43]), whereas research from India frequently prioritized accessible diagnostic tools, particularly respiratory sound analysis and chest X-rays (e.g., Raju et al. [44], Sahu et al. [45], Jayadharshini et al. [37]). In contrast, North American and European research was characterized using large-scale longitudinal cohorts, such as COPDGene and DLCST (utilized by González et al. [46], Tang et al. [47], and Cheplygina et al. [48])—to support comprehensive phenotyping.

The implemented AI technologies spanned several categories. DL models were prevalent, including CNNs and their variants, as well as Long Short-Term Memory (LSTM) networks, which were often applied to sequential data like respiratory sounds. Common ML algorithms were also used, such as Support Vector Machines (SVM), Random Forest (RF), and ensemble/boosting models (e.g., XGBoost, CatBoost). Additionally, LLMs like GPT-4 were tested [36,49].

Reported diagnostic performance varied substantially across studies. Some models reported accuracy in the 80-90% range, such as 80.77% for an LS-SVM [50], 86.59% for a Decision Tree (DT) model [39] 88.7% for a Custom CNN [51], and 89.47% for a 1D CNN [52]. Many studies reported performance metrics exceeding 95%. For example, Gökçen [38] achieved 95.28% accuracy with AdaBoost, Melekoglu et al. [53] reported 96.3% accuracy with a hybrid model on PPG signals, and Koysalya et al. [54] reported 97% accuracy with an XGBoost model on genetic data. Several studies reported exceptionally high performance, including accuracies of 99.62% (VGG16 + LSTM) [55], 99.7% (Quadratic Discriminant Classifier) [34], and 99.9% (Xception model) [40]. Two studies, one by El-Magd, et al. [56] using GoogleNet and another by Mahmood et al. [57] using an RF + MobileNetV2 model, reported 100% accuracy in their respective classification tasks.

Performance was documented using a variety of metrics, including accuracy, AUROC curve, F1-score, precision, and recall. Some studies reported performance on distinct validation sets. For instance, Sun et al. [58] reported an AUC of 0.934 on an internal test set and 0.866 on an external validation dataset, while Cheplygina et al. [48] noted varying AUCs (from 0.79 to 0.956) when transferring models across different datasets.

### 3.3. Outcome Prediction and Prognostic Modeling

A total of 34 studies, shown in Table 2, focused on AI-based prediction of clinical outcomes. The objectives were diverse and included the prediction of exacerbations [59,60,61,62], hospital admission or readmission risk [63], disease progression or severity [64,65], and mortality [46,66,67].

Geographically, outcome prediction research was heavily represented by North America and Europe, likely due to the availability of long-term longitudinal data required for prognostic modeling. Studies from the USA frequently leveraged extensive established cohorts; for instance, González et al. [46] and Young et al. [68] utilized data from the COPDGene study to develop phenotype clustering and progression models. Similarly, European research, particularly from Germany and Spain, utilized multi-center clinical registries. Almeida et al. [65] utilized the German COSYCONET cohort for severity analysis, while Spanish teams, such as Casal-Guisande et al. [69], focused heavily on developing Intelligent Clinical Decision Support Systems (iCDSSs) for mortality prediction using hospital administrative data.

Frequently applied approaches included Artificial Neural Networks (ANNs) [70] including CNNs [46], RF [67], and Gradient-boosting models like GBM [59] and other ensemble models [71] were also represented. Most studies relied on EHRs/clinical variables [59,60,67,71,72], with fewer incorporating medical imaging (CTs) [46,65,66] or respiratory sounds [61]. Collectively, models were developed for both short- and long-term outcomes.

Predictive performance varied substantially. For exacerbation prediction, Wang et al. [60] reported an AUC of 0.90 with an SVM, and Kor et al. [59] achieved an AUC of 0.832 with a GBM. For mortality, Enríquez-Rodríguez et al. [67] reported 99% accuracy using RF, while González et al. [46] achieved an AUROC of 0.72 with CNN. For hospital readmission, Huang et al. [63] reported 77.2% accuracy.

Direct comparisons with conventional indices were rare. One study by Nam et al. [66] evaluated their DL model against the BODE index for survival prediction, finding similar rather than superior results (Time-Dependent AUC 0.87 vs. 0.80). Reporting of key methodological aspects was limited. Nam et al. [66] was one of the few studies to explicitly mention external validation. Interpretability was addressed in just a few cases, such as by Shaikat et al. [72].

### 3.4. Symptom-Based and Monitoring Applications

Six studies focused on AI-driven symptom monitoring and patient management in COPD, shown in Table 3. Geographically, this domain was characterized by exploratory studies from diverse regions rather than large, centralized cohorts. Research from Japan was prominent, with Yamane et al. [73] utilizing tri-axial accelerometer data to recognize activities causing dyspnea and Hirai et al. [74] applying clustering techniques to patient data. In Europe, Amado-Caballero et al. [75] focused on audio cough analysis, while contributions from China and South Korea (e.g., Peng et al. [76], Pant et al. [75]) leveraged IoT platforms and national survey data, respectively.

These studies primarily explored wearable sensor data, cough and respiratory sound analysis, and mobile health platforms to assess symptom burden, daily activity, and early exacerbation detection. ML algorithms such as RF, GB, and DL algorithms such as RNNs were used to analyze time-series data including oxygen saturation, step counts, and respiratory rate. A few studies incorporated NLPs or voice analysis to identify early signs of deterioration.

Despite encouraging results, most studies were exploratory, conducted on small cohorts, and lacked standardized validation frameworks. Broader, multi-center studies integrating multimodal data could strengthen clinical applicability.

**Table 1:** Descriptive table of scoping reviews in the diagnosis category.

Author	Purpose	AI Model	Data Source	Main Result
Lin et al. [41]	Diagnosis	CatBoost, XGBoost, LightGBM, Gradient Boosting Classifier	Electronic Health Records and outpatient medical records	CatBoost was highlighted as the most effective model in terms of accuracy and sensitivity for detecting high-risk populations for COPD.
Heyman et al. [77]	Diagnosis (Early detection, differentiation)	CatBoost, CareNet	Dyspnea patients	CareNet model performed better than CatBoost. Sensitivity: 0.919 (CareNet) vs. 0.871 (CatBoost).
Saad et al. [39]	Diagnosis	DT, SVM, KNN, Naive Bayes, Neural Networks	Pulmonary function tests	DT provided the best results. Accuracy: 0.8659.
Rivas-Navarrete et al. [52]	Diagnosis	1D-CNN, SVM	Cough and breath sounds	1D-CNN provided the best results. Accuracy: 0.8947, Precision: 0.80, Recall: 1.00, F1-score: 0.8889.
Zhang et al. [78]	Diagnosis	GPT-4, Rule-based Classifier, Traditional ML Classifier, ChatGPT, LLaMA3 (8B)	Electronic Health Records	Rule-based and Traditional ML Classifiers performed best. F1-score (Rule-based): 0.9600, F1-score (ML): 0.9600, F1-score (GPT-4): ~0.9444.
Maldonado-Franco et al. [79]	Diagnosis	Neural Networks	Patient records	Accuracy: 0.929, Sensitivity: 0.882, Specificity: 0.943.
Guan et al. [42]	Diagnosis	Gradient Boosting Decision Tree (GBDT), CNN	CT imaging features, lung density parameters, and clinical characteristics	GBDT model (using radiomic, lung density, and clinical data) provided best results. AUC: 0.73, Accuracy: 0.81, Sensitivity: 0.84.
Almeida et al. [80]	Diagnosis	Anomaly Detection, PCA	CT scans	Anomaly scores improved predictive power. Adjusted R^2^: 0.56 (from 0.22), Correlation (Emphysema): 0.66, Correlation (Small Airway Disease): 0.61.
Davies et al. [81]	Diagnosis	CNN	Surrogate data, Photoplethysmography (PPG) Data, Real-World COPD Data	AUC (Surrogate data): 0.75, AUC (Real-world data): 0.63, Accuracy Range: 0.40 to 0.88, AUC (2 cycles): 0.75.
Zhang et al. [49]	Diagnosis	CNN, LSTM, CNN-LSTM, CNN-BLSTM	Audio data	LSTM provided the best results. Accuracy: 0.9882, F1-score: 0.97.
Albiges et al. [82]	Disease classification	RF, SVM, Gaussian Mixture Model (GMM), DT	Audio data	RF provided best results. COPD vs. Healthy: Accuracy: 0.80, F1-score: 0.785. COPD vs. Healthy vs. Pneumonia: Accuracy: 0.70, F1-score: 0.597.
Melekoglu et al. [53]	Diagnosis	SVM, KNN, Ensemble Trees, hybrid models	Photoplethysmography (PPG) signal	Hybrid model (40% features): Sensitivity: 0.942, Accuracy: 0.963. Hybrid model (45% features): AUC: 0.952.
Vollmer et al. [51]	Diagnosis (Case vs. control)	LR, Random Forest Classifier, SGD Classifier, KNN, Decision Tree Classifier, GaussianNB, SVM, Custom CNN, MLP	Patient data	Custom CNN provided the best results. Accuracy: 0.887, AUC: 0.953.
Bracht et al. [83]	Disease differentiation	Random Forest, SVM, Linear Discriminant Analysis	Mass spectrometry analysis of plasma samples	RF presented best results. AUC (Adenocarcinoma vs. COPD): 0.935, AUC (COPD w/ Adenocarcinoma vs. COPD): 0.916.
Joumaa et al. [33]	Disease differentiation	Multinomial Regression, Gradient Boosting, RNN	Patient data from medico-administrative databases	Boosting model results: Recall (Asthma): 0.83, Recall (COPD): 0.64, Precision (Asthma): 0.71, Precision (COPD): 0.66.
Zafari et al. [84]	Diagnosis (Case vs. control)	Multilayer Neural Networks (MLNN), XGBoost	Electronic Health Records	XGBoost provided the best results. Overall Accuracy: 0.86, AUC (Structured data): 0.919, AUC (Text data): 0.882, AUC (Mixed data): 0.932.
Zheng et al. [50]	Diagnosis (Case vs. control)	LS-SVM (linear and polynomial kernels)	Patient data	Both kernels provided optimal results. Linear kernel: Accuracy: 0.8077, AUC: 0.87. Polynomial kernel: Accuracy: 0.8462, AUC: 0.90.
Tang et al. [47]	Diagnosis	ResNet	Chest CT	AUC: 0.86, PPV: 0.847, NPV: 0.755.
González et al. [46]	Diagnosis, staging, and prediction (ARD, mortality)	CNN	Chest Computed Tomography from COPDGene participants	AUC (Mortality): 0.72, AUC (Diagnosis): 0.856, AUC (Exacerbation): 0.64.
El-Magd et al. [56]	Diagnosis (Early detection)	GoogleNet	Sensor data and patient data	Model achieved perfect classification. Accuracy: 1.00, Precision: 1.00, Recall: 1.00, F1-score: 1.00.
Mahmood et al. [57]	Diagnosis	Random Forest, MobileNetV2	Audio data	Accuracy: 1.00, Sensitivity: 1.00, Precision: 1.00, F1-score: 1.00.
Choi et al. [85]	Diagnosis	Modified VGGish, LACM, Grad-CAM	Respiratory sounds	Accuracy: 0.9256, Precision: 0.9281, Sensitivity: 0.9222, Specificity: 0.9850, F1-score: 0.9229, Balanced Accuracy: 0.954.
Brunese et al. [86]	Diagnosis	k-Nearest Neighbors (kNN), SVM, Neural Networks, Logistic Regression	Respiratory sounds	Neural network provided the best results. F1-score: 0.960, Sensitivity: 0.95, Specificity: 0.970.
McDowell et al. [87]	Diagnosis	Generalized Linear Model (GLM), Gradient Boosting Model, ANN, Ensemble Method (ANN/GBM)	Patient serum samples	The ensemble model (ANN/GBM) provided the best results. Mean AUC: 0.93.
Yahyaoui et al. [88]	Diagnosis	SVM, ASVM (Adaptive SVM)	Patient data	Adaptive SVM provided better results than SVM. Accuracy (ASVM): 0.9263, Accuracy (SVM): 0.9059.
Saleh et al. [89]	Diagnosis	DT, NB, Bayesian Networks, Wrapper Methods, Discretization Algorithms	Patient data	Bayesian network with the TAN algorithm performed best. AUC: 0.815.
Rukumani Khandhan et al. [90]	Diagnosis	Inception, ResNet, VGGNet	Respiratory sounds	InceptionNet performed the best. F1-Score: 0.99, Precision: 1.00, Recall: 0.98, Accuracy: 0.99.
Gökçen [38]	Diagnosis	SVM, AdaBoost, RF, J48 Decision Tree	Lung sounds	AdaBoost achieved the highest performance. Accuracy: 0.9528, Sensitivity: 0.9032, Specificity: 0.9987.
Hung et al. [91]	Diagnosis	CNN	Cough sounds	Accuracy: 0.91.
Chawla et al. [92]	Diagnosis	SVM, ResNet50	Chest X-Ray	SVM + ResNet50 model results: Accuracy: 0.93, Precision: 0.94, Recall: 0.928, F1-score: 0.933.
Kousalya et al. [54]	Diagnosis	SVM, LR, DT, KNN, MLP, XGBoost	Patient data including genetic data	XGBoost provided the best results. Accuracy: 0.97, Mean AUC: 0.94, Sensitivity: 0.99.
Wu et al. [36]	Diagnosis	SVM, Random Forest, Deep Neural Networks	CT scans	RF provided the best results. Sensitivity: 0.925, Specificity: 0.902, Accuracy: 0.9149.
Archana et al. [55]	Diagnosis and disease differentiation	VGG16 Model, LSTM	Lung sounds	Overall Accuracy: 0.9962.
Zhu et al. [93]	Disease classification	Bidirectional Gated Recurrent Units (BiGRU), CNN	Respiratory sounds	COPD model performance: Precision: 1.00, Recall: 0.87, F1-score: 0.93. Overall Accuracy: 0.919.
Jayadharshini et al. [37]	Diagnosis and severity assessment	InceptionV3, VGG16, ResNet, DenseNet, XGBoost	Chest X-rays	Severity (XGBoost): Precision: 0.95, Recall: 0.92, F1-score: 0.96. Diagnosis (InceptionV3): Accuracy: 0.9285.
Raju et al. [44]	Disease classification	CNN	Respiratory sounds	COPD Diagnosis results: AUC: 0.98, F1-score: 0.90, Recall: 0.89, Precision: 0.95. Overall Accuracy: 0.93.
Türkçetin et al. [94]	Diagnosis	DenseNet201, VGG16, CNN	CT scans	Accuracy: 0.99, Recall: 0.98, Precision: 1.00, F1-Score: 0.99.
Wang et al. [95]	Diagnosis	Transfer learning	Patient data and Electronic Health Records	AUC: 0.952, Accuracy: 0.905, F1-score: 0.887.
Choudhary et al. [96]	Diagnosis and disease differentiation	Ensemble learning (CNN, XGBoost, RF, SVM, LR)	X-ray images	Ensemble model performed best. Accuracy: 0.948, Sensitivity: 0.936, Specificity: 0.959, AUC: 0.97. (Outperformed standalone CNN Accuracy: 0.902).
Ooko et al. [97]	Diagnosis	TinyML, Synthetic Data model	Synthetic data generated from exhaled breath samples	TinyML model provided the best results. Accuracy: 0.9778.
Rohit et al. [98]	Diagnosis and disease differentiation	BiLSTM	Respiratory sounds	Accuracy: 0.96, F1-score: 0.96.
Islam et al. [99]	Diagnosis and disease differentiation	LR, Random Forest, GB, SVM, Naive Bayes, ANN, CNN, 1D-CNN, LSTM	Respiratory sounds + clinical data	Respiratory dataset (ANN): Accuracy: 0.485, Precision: 0.50, F1-score: 0.465, Recall: 0.485. ICHBI dataset (1D-CNN): Accuracy: 0.92, Precision: 0.89, Recall: 0.91, F1-score: 0.93.
Ikechukwu et al. [100]	Diagnosis	ResNet50, Xception, Transfer Learning	Chest X-Rays	Highest performance in Lung Nodule detection. Accuracy: 0.930, Precision: 0.97, Recall: 0.965, F1-score: 0.967.
Jenefa et al. [101]	Diagnosis	CNN-LSTM	Lung function measurements, clinical history, and image data	Accuracy: 0.963, Precision: 0.948, Recall: 0.972, F1-score: 0.959.
Moran et al. [40]	Diagnosis	Xception, VGG-19, InceptionResNetV2, DenseNet-121	ECG Signals	Xception Model provided the best results. Accuracy: 0.999, Sensitivity: 0.996.
Anupama et al. [102]	Diagnosis	CNN	Lung sounds	Accuracy: 0.833.
Sahu et al. [45]	Diagnosis and disease differentiation	1D-CNN (Adam and RMSprop optimizers)	Respiratory sounds	1D-CNN with Adam optimizer performed best. Accuracy: 0.94, Precision: 0.90, Recall: 0.86, F1-score: 0.88.
Ooko et al. [103]	Diagnosis	TinyML (NN, K-means)	Exhaled breath data	Validation Accuracy: 0.953.
Sanjana et al. [104]	Diagnosis	Convolutional Recurrent Neural Networks (CRNN)	Lung sounds	CRNN-BiLSTM provided the best results. Accuracy: 0.98601, F1-score: 0.99, Recall: 0.98.
Jha et al. [105]	Diagnosis (Early detection)	1D-CNN (Adam and RMSprop optimizers)	Respiratory sounds	1D-CNN with Adam optimizer performed best. Accuracy: 0.94, Precision: 0.90, Recall: 0.86, F1-score: 0.88.
Mridha et al. [106]	Diagnosis	CNN	Respiratory sounds	Accuracy: 0.95, AUC: 1.00.
Ikechukwu et al. [107]	Diagnosis	ResNet50	Chest X-Ray	Pneumothorax case provided best results. Accuracy: 0.986, Precision: 0.994, Recall: 0.986, F1-score: 0.973.
T. Ha et al. [108]	Diagnosis	Random Forest, CNN	Respiratory sounds	Accuracy: 0.9604, Recall: 0.9847, Precision: 0.9905, F1-Score: 0.9775, Specificity: 0.9846.
Dhar [109]	Diagnosis	XGBoost, Extra Trees, Random Forest, GB, LR, SVC, KNN, NuSVC	Dielectric and demographic data	Ensemble learning model results: Accuracy: 0.9820, Precision: 0.98, Recall: 0.96, F1-score: 0.9667, AUC: 0.9912.
Khade [110]	Diagnosis and disease differentiation	Deep CNN	Breathing patterns and chest X-ray pictures	Accuracy: 0.98, Precision: 0.99, Recall: 0.98, F1-score: 0.98.
Fang et al. [43]	Diagnosis	DSA-SVM	Electronic Health Records	Accuracy: 0.951, Recall: 0.9793, F1-score: 0.9771.
Li et al. [111]	Diagnosis	CNN, Fuzzy decision trees	Respiratory sounds	CNN provided high classification accuracy. Fuzzy decision tree provided interpretable predictions. Confidence Level: 0.84.
Bulucu et al. [112]	Diagnosis	Recurrent Trend Predictive Neural Network	E-Nose sensor data	Overall Accuracy: 0.97, Recall: 0.9896, Specificity: 0.9455, F1-score: 0.9726, MCC: 0.9416.
Aulia et al. [113]	Diagnosis	Graph Convolutional Network, PCA	Exhaled breath data	Accuracy: 0.975, Precision: 0.972, Recall: 0.974, F1-score: 0.975.
Amudala Puchakayala et al. [114]	Diagnosis	CatBoost	CT scans	Standard-Dose CT: AUC: 0.90, PPV: 0.83, NPV: 0.83. Low-Dose CT: AUC: 0.87, PPV: 0.79, NPV: 0.80. Combined CT + Clinical: AUC: 0.88, PPV: 0.79, NPV: 0.80.
Zhang et al. [115]	Diagnosis	LASSO regression model, SVM-RFE	Gene expression data	SLC27A3 and STAU1 achieved highest AUCs. AUC (SLC27A3): 0.900, AUC (STAU1): 0.971.
Sun et al. [58]	Diagnosis	ResNet18	Chest CT scans and clinical data	AUC (Internal test set): 0.934, AUC (External validation): 0.866.
Zhang et al. [116]	Diagnosis	Bagged DT	Respiratory signals	Accuracy: 0.933.
Wu et al. [117]	Diagnosis	Random Forest, DT, KNN, Linear Discriminant Analysis, AdaBoost, DNN	Wearable device data	DNN performed the best. Accuracy: 0.914, F2-score: 0.914, AUC: 0.9886, Sensitivity: 0.877, Specificity: 0.955, Precision: 0.955.
Srivastava et al. [118]	Diagnosis	CNN	Respiratory sounds	MFCC data (post-augmentation): Sensitivity: 0.92, Specificity: 0.92, ICBHI Score: 0.92. Mel-Spectrogram data: Sensitivity: 0.73, Specificity: 0.91, ICBHI Score: 0.82.
Zakaria et al. [119]	Diagnosis (differentiation, case vs. control)	ResNet50, ResNet101, ResNet152	Respiratory sounds	ResNet50 provided best accuracy/time trade-off. Accuracy: 0.9037.
Bodduluri et al. [120]	Diagnosis and classification	Fully Convolutional Network (FCN), Random Forest Classifier (RFC)	Spirometry data	Both models were promising. FCN: AUC: 0.80, F1-score: 0.79. RFC: AUC: 0.90, F1-score: 0.76.
Ma et al. [121]	Diagnosis	LR, KNN, SVM, DT, MLP, XGboost	Clinical and genetic data	XGBoost provided the best results. AUC: 0.94, Accuracy: 0.91, Precision: 0.94, Sensitivity: 0.94, F1-score: 0.94, MCC: 0.77, Specificity: 0.84.
Naqvi et al. [34]	Diagnosis and disease differentiation	SVM, Quadratic Discriminant Classifier, KNN, RF, Rule-based Systems	Lung sounds	Quadratic Discriminant Classifier performed best. Accuracy: 0.997, TPR (Recall): >0.99.
Basu et al. [122]	Diagnosis and disease differentiation	Deep Neural Network	Respiratory sounds	Overall Accuracy: 0.9567, Precision: 0.9589, Recall: 0.9565, F1-score: 0.9566. COPD-specific: Precision: 1.0, Recall: 0.91, F1-score: 0.95.
Altan et al. [123]	Diagnosis (Early detection)	Deep Belief Network	Lung sounds	Accuracy: 0.9367, Sensitivity: 0.91, Specificity: 0.9633.
Spathis et al. [35]	Diagnosis and disease differentiation	Random Forest, NB, LR, NN, SVM, KNN, DT	Patient data	RF model provided the best results. Precision: 0.977.
Xu et al. [124]	Diagnosis (Symptom detection)	ANN	Electronic Health Records	Accuracy: 0.8645, F1-score: 0.8293.
Haider et al. [125]	Diagnosis (Case vs. control)	SVM, KNN, LR, DT, Discriminant Analysis	Respiratory sounds	LR and SVM (linear and quadratic) performed best. Accuracy: 1.00, Sensitivity: 1.00, Specificity: 1.00, AUC: 1.00.
Gupta et al. [126]	Diagnosis and disease differentiation	KNN, SVM (Linear), Random Forest, Decision Tree	Chest CT scans	IGWA with KNN classifier achieved highest accuracy. Accuracy: 0.994.
Badnjevic et al. [127]	Diagnosis and disease differentiation	ANN, Fuzzy Logic	Patient data and clinical data	Sensitivity: 0.9622, Specificity: 0.9871.
Windmon et al. [128]	Diagnosis and disease differentiation	Random Forest	Cough recordings	Lvl 1 (Disease vs. Control): AUC: 0.83, Accuracy: 0.8067, Sensitivity: 0.80, Specificity: 0.82. Lvl 2 (COPD vs. CHF): AUC: 0.80, Accuracy: 0.7805, Sensitivity: 0.82, Specificity: 0.75.
Pizzini et al. [129]	Diagnosis (Case vs. control)	Random Forest	Breath samples	AUC: 0.97, Sensitivity: 0.78, Specificity: 0.91, PPV: 0.86, NPV: 0.86.
Cheng et al. [130]	Diagnosis	SPADE	Clinical data	Model demonstrated high sensitivity and specificity. (No numerical values provided).
Cheplygina et al. [48]	Diagnosis (Case vs. control)	Transfer learning	CT scans	Best performance on Frederikshavn dataset. AUC: 0.938–0.953. AUC (COPDGene2): 0.956, AUC (COPDGene1): 0.917, AUC (DLCST): 0.79.

**Table 2:** Descriptive table of scoping reviews in the outcome category.

Author	Purpose	AI Model	Data Source	Main Result
Almeida et al. [65]	Severity	Self-supervised DL anomaly detection	Paired inspiratory/expiratory CT and clinical data (COPDGene, COSYCONET)	AUC (COPDGene): 0.843, AUC (COSYCONET): 0.763.
Wang et al. [71]	Risk Prediction	CatBoost, NGBoost, XGBoost, LightGBM, RF, SVM, LR	Clinical data	CatBoost model performed best. AUC: 0.727, F1-score: 0.425, Accuracy: 0.736.
Dogu et al. [70]	Length of hospital stay	SBFCM, ANN	Clinical findings, socio-demographic information, comorbidities, medical records	Accuracy: 0.7995 (outperformed other models).
González et al. [46]	Detect/stage COPD, predict ARD events & mortality	CNN	Chest CT (COPDGene)	AUROC (Mortality): 0.72, AUROC (Diagnosis): 0.856, AUROC (Exacerbation): 0.64.
Huang et al. [63]	Hospital readmission	NLP, DT	Patient discharge reports	Accuracy: 0.772 (in terms of readmission risk).
Zheng et al. [64]	Severity	HFL-COPRAS	Patient data	Sensitivity analysis showed rankings can vary but the method remains robust.
Baechle et al. [131]	Hospital readmission	NB, RF, SVM, KNN, C4.5, Bagging, Boosting	Patient discharge reports	RF achieved highest AUC: 0.657. Naïve Bayes had lowest mean misclassification cost.
Wang et al. [132]	Treatment	Association Rules, Cluster Analysis, Complex Network Analysis	Prescription data	Identified key traditional Chinese medicines and associations for holistic treatment.
Jayadharshini et al. [37]	Severity, Diagnosis	InceptionV3, VGG16, ResNet, DenseNet, XGBoost	Chest X-rays	Severity (XGBoost): Precision: 0.95, Recall: 0.92, F1-score: 0.96. Diagnosis (InceptionV3): Accuracy: 0.9285.
Shaikat et al. [72]	Severity, Quality of life	XGBoost, RF, XAI	Patient data	Severity (XGBoost): Accuracy: 0.9955. Quality of Life (RF): MSE: 94.95, MAE: 7.06.
Nam et al. [66]	Survival	CNN	Post-bronchodilator spirometry and chest radiography	TD AUC (DLSP CXR): 0.73, TD AUC (DLSP integ): 0.87. (Outperformed FEV1).
Hasenstab et al. [133]	Mortality/Severity	CNN	CT images	AUC (%EM): >0.82 (GOLD 1-3), AUC (%EM): >0.92 (GOLD 4).
Hussain et al. [134]	Severity	RF, SVM, GBM, XGBoost, KNN, SVE	Patient data	SVE performed best. Accuracy: 0.9108, Precision: 0.9077, Recall: 0.9136, F-measure: 0.9107, AUC: 0.9687.
Peng et al. [135]	Severity	DT	Radiology reports	Sensitivity: 0.869. Model with %LAV-950 and AWT3-8 was superior to %LAV-950 alone (AUC: 0.92 vs. 0.79).
Altan et al. [136]	Severity	DELM	Lung sounds	COPD0: Accuracy: 0.9333. COPD1: Accuracy: 0.9003. COPD2: Accuracy: 0.9523. COPD3: Accuracy: 0.8571. COPD4: Accuracy: 0.9902.
Young et al. [68]	Progression	Clustering	COPDGene	Identified two distinct COPD subtypes: Tissue → Airway (70%) and Airway → Tissue (30%).
Goto et al. [137]	Hospital readmission	LR, Lasso, DNN	Patient data	Sensitivity (LR): 0.75 vs. Sensitivity (DNN): 0.67. Specificity (Lasso): 0.51 vs. Specificity (LR): 0.37.
Orchard et al. [138]	Hospital admission risk	MT-NN, SVM, RF	Trial data	Multi-task neural nets performed best for 24-hour admission prediction. AUC: 0.74.
Swaminathan et al. [139]	Triage	SVM, RF, NB, LR, KNN, GBRF, ET	Patient data	Logistic Regression and Gradient Boosted Random Forest provided the best accuracy.
Casal-Guisande et al. [69]	Mortality	SqueezeNet	Patient data	AUROC: 0.85.
Casal-Guisande et al. [140]	Exacerbation characterization	k-prototypes, RF	Patient data	Identified four unique clusters. AUROC: 0.91.
López-Canay et al. [141]	Hospital readmission	RF, NB, MLP, Fuzzy Logic	Patient data	AUC: ~0.80, Sensitivity: 0.67, Specificity: 0.75.
Jeon et al. [142]	Severity	1D-Transformer, MLP, LR	Clinical data with spirometry images	Transformer + MLP outperformed LR. AUROC (Mod-Sev): 0.755 vs. 0.730. AUROC (Severe): 0.713 vs. 0.675.
Pegoraro et al. [143]	Exacerbation prediction	HMM	Remote monitoring device data	HMM improved detection of pre-exacerbation periods. Sensitivity: up to 0.768.
Atzeni et al. [144]	Exacerbation prediction	k-means, LR, RF, XGBoost	Air quality, health records, lifestyle info	RF performed best. Cluster 1: AUC: 0.90, AUPRC: 0.70. Cluster 2: AUC: 0.82, AUPRC: 0.56.
Wu et al. [145]	Exacerbation prediction	RF, DT, LDA, AdaBoost, DNN	Lifestyle, environmental, clinical, wearable, interview data	RF model performed best. Accuracy: 0.914, Precision: 0.686, F1-score: 0.680.
Bhowmik et al. [146]	Exacerbation prediction	STAIN, CNN, RNN, CRNN	Audio files	STAIN model performed best. Accuracy: 0.9340, Sensitivity: 0.9270, Specificity: 0.9420, MCC: 0.8691.
Vishalatchi et al. [147]	Exacerbation prediction	NLP, RF, LR, NB, SVM, DT, DNN	Electronic Health Records, Clinical Notes	RF model performed best. Accuracy: 0.80, F1-Score: 0.73.
Kor et al. [59]	Exacerbation prediction	SVM, RF, GBM, XGB	Clinical Data	GBM model performed best. AUC: 0.832, Sensitivity: 0.7941, Specificity: 0.7794, PPV: 0.6429.
Wamg et al. [60]	Exacerbation prediction	RF, SVM, LR, KNN, NB	Electronic Health Records	SVM model performed best. AUROC: 0.90, Sensitivity: 0.80, Specificity: 0.83, PPV: 0.81, NPV: 0.85.
Fernandez-Granero et al. [61]	Exacerbation prediction	Random Forest	Respiratory sounds	Accuracy: 0.878, Sensitivity: 0.781, Specificity: 0.959, PPV: 0.941, NPV: 0.839, F1-score: 0.80, MCC: 0.80.
Shah et al. [62]	Exacerbation prediction	LR, SVM, DT, KNN	Vital signs, symptoms, medication data	LR (using vital signs) performed best. Mean AUC: 0.682.
Enríquez-Rodríguez et al. [67]	Mortality	RF, PLS, KNN	Clinical data, biological samples, follow-up data, comorbidity	RF performed best. Accuracy: 0.99.
Pinheira et al. [148]	Length of hospital stay	CNN	Patient Data	AUC (6-day threshold): 0.77, AUC (10-day threshold): 0.75.

**Table 3:** Descriptive table of scoping reviews in the symptoms category.

Authors	Purpose	AI Model	Data Source	Main Result
Pant et al. [75]	Predict smoking status	RF, DT, Gaussian Naive Bayes, KNN, AdaBoost, MLP, TabNet, ResNN	Demographic, behavioral, and clinical data	ResNN outperformed other models. (Metrics: AUROC, Sensitivity, Specificity, F1-score).
Amado-Caballero et al. [149]	Analyze cough patterns	CNN	Audio data	Distinctions in cough patterns were observed between COPD and other respiratory pathologies.
Yamane et al. [73]	Recognize activities causing dyspnea	RF	Tri-axial accelerometer	The wrist + hip classifier successfully recognized most daily activities that caused shortness of breath.
Weikert et al. [150]	Analyze/quantify airway wall thickness	3D U-Net	Chest CT	Airway centerline detection: Sensitivity: 0.869. Airway wall segmentation: Dice score: 0.86.
Peng et al. [76]	Remote monitoring	LLM	Sensor data	Average model Accuracy: 0.74. The LLM generated short, interpretable rules despite data variations.
Hirai et al. [74]	Differentiate ACOS from asthma/COPD	k-means	Patient data	Identified 4 biological clusters, including a distinct cluster for Asthma-COPD Overlap (ACOS) patients.

## 4. Discussion

### 4.1. Summary of Findings by Category

Our review mapped a substantial body of literature, confirming that AI in COPD is an exceptionally active field with a high volume of publications requiring continuous review [26]. A key contribution of this scoping review is the provision of a global, structured overview of this broad landscape, whereas other recent valuable reviews have necessarily focused on specific domains, such as meta-analyses of CT-based diagnosis [27] or prognostic models [28]. Our findings reveal a clear concentration of research in three primary domains, which provides a holistic framework: (1) diagnostic applications, (2) outcome prediction and prognostic modeling, and (3) symptoms and phenotype analysis. The state of the evidence within each of these categories will be summarized in the sections that follow.

#### 4.1.1. AI in COPD Diagnosis

The most prominent application of AI identified in this review was for the diagnosis and detection of COPD. This area is characterized by a wide variety of advanced models and data sources, often reporting high-performance metrics. ML models, particularly tree-based ensembles, have proven effective when applied to large clinical datasets. For instance, Lin et al. [41] demonstrated that the CatBoost model was highly effective for identifying high-risk populations from EHRs.

DL algorithms, more specifically CNNs, have been extensively used for analyzing medical imagery. Studies conducted by Guan et al. and Tang et al. [42,47] showcased the power of CNNs in accurately diagnosing COPD from chest CT scans. The application of AI extends beyond traditional data types; researchers have successfully used audio data, with Rivas-Navarrete et al. [52] employing a 1D-CNN to detect COPD from cough and breath sounds with 89.5% accuracy.

Several studies reported exceptionally high, near-perfect diagnostic accuracy. El-Magd et al. [56], for example, achieved an accuracy of 100% using GoogleNet architecture with sensor data, while Mahmood et al. [57] reported similar success with a hybrid RF + MobileNetV2 model on audio recordings. The field also shows rapid innovation through the adoption of emerging technologies, such as the use of LLMs like GPT-4, which Zhang et al. [78] found to achieve an F1-score of 94.4% on EHRs.

Collectively, these findings illustrate a strong trend: researchers are successfully applying sophisticated algorithms to diverse data, from structured EHRs and images to unstructured audio and sensor data, to achieve rapid, accurate, and non-invasive COPD detection.

#### 4.1.2. AI in COPD Outcome Prediction

Beyond diagnosis, a significant body of research focuses on leveraging AI to predict critical clinical outcomes, which is essential for proactive disease management and resource allocation. A primary target for prediction is AE-COPD, a major cause of hospitalization and disease progression. Researchers have employed a variety of models to tackle this, with Wang et al. [60] evaluating five different ML algorithms on electronic medical records. Their findings showed that an SVM achieved the best overall performance, with a high AUC of 0.90 and strong sensitivity (0.80) and specificity (0.83). Similarly, Kor et al. [59] tested four models on clinical data and found that a GBM provided the most robust results in predicting AECOPD, achieving an AUC of 0.832.

The use of non-traditional data for outcome prediction is a notable trend. Fernandez-Granero et al. [61] pioneered an approach using a decision tree forest classifier on daily home-recorded respiratory sounds. Their model was able to predict exacerbations with an average of 4.4 days’ notice, demonstrating an impressive accuracy of 87.8% and a high specificity of 95.9%. This highlights the potential for remote monitoring systems to provide early warnings for deteriorating patient conditions.

AI has also been applied to forecast other crucial long-term outcomes. For instance, González et al. [46] utilized a CNN on chest CT data from the large COPDGene cohort to predict mortality, achieving a respectable AUROC of 0.72. Predicting hospital readmission is another key application, with Huang et al. [63] using a novel combination of text mining on patient discharge reports and a decision tree algorithm to identify patients at high risk of 30-day readmission, achieving an accuracy of 77.2%.

Furthermore, AI models have been developed to assess disease severity, moving beyond simple prediction to a more nuanced understanding of a patient’s condition. Almeida et al. [65] developed a self-supervised DL model that could detect anomalies in paired inspiratory/expiratory CT scans, demonstrating its effectiveness with an AUC of 84.3% in the COPDGene dataset. Other studies, such as one by Dogu et al. [70], have focused on operational outcomes, using an integrated approach of statistical-based fuzzy cognitive maps and ANNs to predict the length of hospital stay with nearly 80% accuracy.

Collectively, these studies show that AI can effectively leverage diverse data sources, from structured clinical data and imaging to unstructured text and audio signals, to build powerful predictive tools for nearly every stage of the COPD management pathway.

#### 4.1.3. AI in COPD Symptom Analysis

A more nascent but equally important application of AI is the analysis of specific COPD symptoms and the identification of distinct patient subgroups. This research moves beyond simple diagnosis towards a more nuanced, personalized understanding of the disease. AI has been effectively used for remote monitoring of symptoms, a critical component of modern chronic disease management. For instance, Yamane et al. [73] employed a RF model with data from a tri-axial accelerometer to successfully recognize daily activities that cause shortness of breath in patients. In a similar vein, Amado-Caballero et al. [149] utilized a CNN to analyze audio recordings, finding distinct cough patterns that could differentiate COPD patients from those with other respiratory conditions.

Beyond symptom tracking, AI is being used to quantify physiological markers and stratify patients. Weikert et al. [150] developed a 3D U-Net model to automatically quantify airway wall thickness from chest CT scans, achieving a high Dice score of 0.86, which is crucial for assessing disease progression. Furthermore, researchers are using unsupervised learning to uncover hidden patient phenotypes. Hirai et al. [74] applied the k-means clustering algorithm to patient data and successfully identified four distinct biological clusters, including a clear asthma-COPD overlap phenotype. This approach is vital for tailoring treatments to specific patient subgroups.

Finally, researchers are exploring behavioral and predictive analytics, such as the work by Pant et al. [75], who found that a ResNN outperformed other models in predicting smoking status based on demographic and clinical variables. These studies collectively exemplify AI’s growing role in moving beyond diagnostics to enable continuous remote monitoring and advance personalized medicine by stratifying patients based on their unique clinical and behavioral characteristics.

Despite these promising results across diagnostic, prognostic, and symptom-analysis applications, the consistently high performance metrics reported in many studies may reflect methodological limitations rather than true model robustness, underscoring the need for careful critical appraisal of the literature.

#### 4.1.4. Cross-Study Methodological Concerns

Although the studies included in this review frequently report high or even near-perfect performance metrics, these results must be interpreted with caution. A substantial proportion of the identified models were trained on small or single-center datasets, a scenario that increases the risk of overfitting and limits the reliability of reported accuracy. In several cases, methodological descriptions were insufficient to rule out forms of data leakage—such as improper train-test splits, patient overlap across folds, or preprocessing steps applied before dataset partitioning—which can artificially inflate performance well beyond what would be achievable in real-world practice.

In addition, many studies relied heavily on accuracy as the primary metric, despite its well-recognized limitations in imbalanced clinical datasets. Only a minority of papers reported calibration, class-wise performance, or clinically relevant measures such as decision-curve analysis. Very few studies compared AI models against established clinical baselines—such as spirometric thresholds, radiologist assessment, or GOLD categorization—making it difficult to contextualize the practical value of the proposed models.

The scarcity of prospective validation, limited reporting of demographic representativeness, and near-absence of fairness or explainability analyses further highlight that the field remains in an early stage of maturity. While the literature demonstrates remarkable innovation and technical sophistication, these methodological weaknesses collectively undermine the robustness and trustworthiness of the evidence base and caution against assuming that current AI systems are ready for clinical deployment.

### 4.2. Limitations of This Review

This review has several limitations that should be acknowledged. Although the number of eligible studies was substantial, the marked methodological heterogeneity across publications precluded quantitative synthesis or meta-analysis. Additionally, despite the use of comprehensive and systematic search strategies, some relevant studies may have been inadvertently omitted, particularly those with insufficient methodological detail in titles or abstracts. Review papers and preprints were excluded a priori to ensure methodological rigor; however, exploratory searches suggested that existing abstracts mentioning both COPD and artificial intelligence were scarce and of limited clinical relevance. Importantly, the methodological heterogeneity across studies—including inconsistent reporting of preprocessing pipelines, training procedures, and validation strategies—limits the reliability of cross-study comparisons and may partially explain the implausibly high accuracies observed in several publications.

A further and critical limitation, evident from the results of this review, is the pervasive lack of external validation for the AI models identified. Most included studies reported performance metrics derived from internal validation, such as train-test splits on a single institutional dataset. As noted in the results, very few studies [48,58] explicitly stated that their models were tested on independent, external cohorts from different institutions or patient populations. This absence of external validation severely limits the generalizability of the reported findings. Models may demonstrate high accuracy due to overfitting to a specific dataset’s characteristics (e.g., patient demographics, CT scanner protocols), and their performance in a broader, real-world clinical setting remains unproven and likely lower than reported.

The search strategy was restricted to English-language publications, which introduces a potential language bias and may limit the generalizability of findings. Furthermore, given the exploration and heterogeneous nature of the included literature, a formal risk-of-bias assessment tool (e.g., PROBAST or QUADAS-2) was not applied. Similarly, no formal inter-rater reliability statistics were calculated for study screening or data extraction. Although discrepancies between reviewers were infrequent and resolved by consensus, this remains a methodological limitation.

Despite extending the search across multiple major databases, including PubMed, Scopus, IEEE Xplore, and Google Scholar, the number of eligible studies remained low. This highlights both the limited availability of robust, clinically validated AI applications in COPD and the urgent need for larger, multi-center, and genotype-diverse prospective studies to strengthen the evidence base and support clinical translation.

Additionally, demographic variables such as age, sex distribution, disease severity, and geographic origin were reported inconsistently across studies, limiting meaningful comparison and hindering assessment of model fairness and generalizability. Studies focusing primarily on other illnesses were excluded unless COPD-specific results were provided, a necessary restriction to maintain relevance but one that may have overlooked potentially transferable AI methodological insights. These limitations underscore opportunities to improve AI learning: future models would benefit from training on diverse, well-annotated populations, integrating multimorbidity profiles common in COPD, and adopting explainable AI approaches that enhance transparency and clinical trustworthiness.

### 4.3. Future Research Directions

Moving forward, the field would benefit from systematic efforts to address the methodological weaknesses identified in the current literature, including improved reporting standards, explicit safeguards against data leakage, rigorous calibration and fairness analyses, and direct comparisons against clinical benchmarks.

To translate the promise of AI into tangible clinical benefits for COPD patients, future research must prioritize the transition from theoretical models to real-world application. The current reliance on retrospective, single-center data necessitates a shift towards prospective, multi-center clinical trials, including Randomized Controlled Trials (RCTs), to validate the efficacy and generalizability of these AI tools. Concurrently, the “black box” nature of many complex algorithms, which hinder clinical trust, must be addressed by embracing Explainable AI (XAI). By integrating techniques like SHapley Additive exPlanations (SHAP) and Local Interpretable Model-agnostic Explanations (LIME), researchers can make model predictions transparent and interpretable, which is crucial for their adoption by healthcare providers who need to understand the reasoning behind an AI-driven recommendation.

Furthermore, the foundation of future research must be strengthened through improved data practices and methodological rigor. The complexity of COPD requires moving beyond siloed information to develop holistic, multi-modal AI models that integrate diverse data streams, including imaging, EHRs, genomics, and patient-generated data from wearables. This will enable a more precise and personalized approach to patient care. To accelerate progress and ensure reproducibility across the field, a collaborative effort is needed to establish standardized reporting guidelines (such as CONSORT-AI and STARD-AI), create public benchmark datasets, and promote the adoption of FAIR (Findable, Accessible, Interoperable, and Reusable) data principles. These foundational steps are essential for building a more robust, collaborative, and impactful research ecosystem.

## 5. Conclusions

This scoping review shows that AI is rapidly evolving and holds strong potential across the clinical management of COPD, from improving diagnostic accuracy to supporting outcome prediction and enabling remote symptom monitoring. These applications collectively point toward more proactive, personalized, and data-driven care. However, several challenges remain, including limited external validation of existing models, heterogeneous study methodologies, and inconsistent demographic reporting, all of which constrain the generalizability and clinical readiness of current AI approaches.

Looking ahead, progress will depend on conducting prospective multi-center studies, adopting explainable and multimodal AI approaches, and increasing access to high-quality and diverse benchmark datasets. Integrating multimorbidity profiles, improving demographic representation, and strengthening regulatory guidance will also be essential for fostering ethical and trustworthy implementation. Addressing these challenges will help ensure that advances in AI translate into meaningful improvements for patients and healthcare systems.

## Data Availability

The original contributions presented in this study are included in the article.

## Abbreviations

The following abbreviations are used in this manuscript:

%EM	Percent Emphysema
%LAV-950	Percentage of Low Attenuation Volume at -950 Hounsfield units
ACOS	Asthma-COPD Overlap Syndrome
AE-COPD	Acute Exacerbation of COPD
AI	Artificial Intelligence
ANN	Artificial Neural Network
ARD	Acute Respiratory Disease
ASVM	Adaptive Support Vector Machine
AUC/AUROC	Area Under the (Receiver Operating Characteristic) Curve
AUPRC	Area Under the Precision-Recall Curve
BiGRU	Bidirectional Gated Recurrent Units
BiLSTM	Bidirectional Long Short-Term Memory
BODE	Body-mass index, airflow Obstruction, Dyspnea, and Exercise capacity
CDSS	Clinical Decision Support Systems
CHF	Congestive Heart Failure
CNN	Convolutional Neural Network
COPD	Chronic Obstructive Pulmonary Disease
CRNN	Convolutional Recurrent Neural Network
CT	Computed Tomography
DL	Deep Learning
DLSP	Deep Learning-based Survival Prediction
DNN	Deep Neural Network
DT	Decision Tree
EHR	Electronic Health Records
FCN	Fully Convolutional Network
FEV1	Forced Expiratory Volume in 1 s
GB/GBC	Gradient Boosting/Gradient Boosting Classifier
GBDT	Gradient Boosting Decision Tree
GBM	Gradient Boosting Machine
GLM	Generalized Linear Model
GMM	Gaussian Mixture Model
GOLD	Global Initiative for Chronic Obstructive Lung Disease
GPT-4	Generative Pre-trained Transformer 4
Grad-CAM	Gradient-weighted Class Activation Mapping
HFL-COPRAS	Hesitant Fuzzy Linguistic COmplex PRoportional ASsessment
ICBHI	International Conference on Biomedical and Health Informatics dataset
KNN/kNN	K-Nearest Neighbors
LACM	Light Attention Connected Module
LIME	Local Interpretable Model-agnostic Explanations
LLM	Large Language Model
LR	Logistic Regression
LS-SVM	Least-Squares Support Vector Machine
LSTM	Long Short-Term Memory
MAE	Mean Absolute Error
MCC	Matthews Correlation Coefficient
MFCC	Mel-Frequency Cepstral Coefficients
ML	Machine Learning
MLNN	Multilayer Neural Networks
MLP	Multilayer Perceptron
MLR	Multivariable Logistic Regression
MSE	Mean Squared Error
NB	Naive Bayes
NGBoost	Natural Gradient Boosting
NLP	Natural Language Processing
NPV	Negative Predictive Value
PCA	Principal Component Analysis
PPG	Photoplethysmography
PPV	Positive Predictive Value
PRM	Parametric Response Mapping
R^2^	R-squared
ResNN	Residual Neural Network
RF	Random Forest
RFC	Random Forest Classifier
RNN	Recurrent Neural Network
SBFCM	Subtractive-Based Fuzzy C-Means
SGD	Stochastic Gradient Descent
SHAP	SHapley Additive exPlanations
STAIN	Spatio-Temporal Artificial Intelligence Network
SVE	State Vector Estimation
SVM	Support Vector Machine
TD AUC	Time-Dependent Area Under the Curve
TinyML	Tiny Machine Learning
TPR	True Positive Rate
XAI	Explainable Artificial Intelligence
XGBoost	Extreme Gradient Boosting
